# A treatment algorithm for couples with unexplained infertility based on sperm chromatin assessment

**DOI:** 10.1007/s10815-018-1270-x

**Published:** 2018-07-28

**Authors:** C. L. O’Neill, A. Parrella, D. Keating, S. Cheung, Z. Rosenwaks, G. D. Palermo

**Affiliations:** The Ronald O. Perelman and Claudia Cohen Center for Reproductive Medicine, Weill Cornell Medicine, 1305 York Avenue, Suite Y-720, New York, NY 10021 USA

**Keywords:** Sperm DNA fragmentation, ICSI, TESE, IVF, Unexplained infertility

## Abstract

**Objective:**

To design a reproductive treatment algorithm based on the sperm DNA fragmentation (SDF) for couples with unexplained infertility following a poor intrauterine insemination (IUI) outcome.

**Design:**

Couples that failed IUI with no apparent reproductive issue in both partners were allocated to diverse reproductive treatments on the basis of SDF.

**Setting:**

Reproductive medical center in an academic setting.

**Patient(s):**

Over 4 years, couples with an unexpected poor IUI outcome and no apparent female or male partner reproductive issues were recruited.

**Intervention(s):**

IUI, IVF, and ICSI were performed in the standard fashion following sperm SDF assays.

**Main outcomes measure(s):**

Fertilization rate, implantation rate, pregnancy characteristics, and delivery rates.

**Result(s):**

A total of 354 couples with unexplained infertility and normal semen parameters underwent 1133 IUI cycles. Clinical pregnancy rate (CPR) with IUI at our center in an age-matched cohort is 23.9% while the study cohort had 1.8%. Following SDF assessment, couples with failed IUI attempts but normal SDF (SCSA 9.8 ± 4.6%; TUNEL 11.8 ± 6.2%) underwent IVF with a CPR of 12.7%; those with abnormal SDF underwent ICSI with ejaculated spermatozoa, resulting in a CPR of 18.7%. This group included couples with normal SDF that had failed IVF. Couples with abnormal SDF that failed ICSI with ejaculated spermatozoa achieved a CPR of 31.0% with surgically retrieved spermatozoa.

**Conclusion(s):**

Couples with unexplained infertility that present with unexpectedly poor IUI outcomes can be funneled into a treatment algorithm guided by the integrity of the sperm genome for higher chances of pregnancy using an alternate method of insemination.

## Introduction

Infertility is seen in 15% of couples of reproductive age. Of those that seek treatment, roughly 60% can be identified as having a male and/or female factor affecting their reproductive ability [1]. In the event that couples fail to achieve a pregnancy despite a young female partner displaying a normal ovulation profile with patent tubes and a male partner with adequate semen parameters, the diagnosis of unexplained infertility is applied. This situation is observed in up to 30% of couples unable to reproduce [2]. In these couples, the most cost-effective and conservative method to begin treatment is through superovulation in combination with timed intercourse or intrauterine insemination (IUI) [3]. However, in cases where a couple fails to achieve pregnancy without obvious reasons, the reproductive physician is left only with the option to empirically adopt another treatment. A recent systematic review on the treatment of couples with unexplained infertility concluded that the accrued data was not compelling enough to point to any specific treatment modality to significantly increase their chances of pregnancy but suggested that these couples should be individually assessed and treatment should be based on their unique reproductive profile [2]. A plausible option would be to proceed with in vitro fertilization (IVF) or intracytoplasmic sperm injection (ICSI), which would possibly unveil any issue with oocyte maturity or fertilization competence.

A first line of screening for the male partner is the semen analysis, according to the most recently revised criteria [4], that often is incapable of generating information on the embryonic competence of the male gamete [5]. Many assays have been utilized to supplement the standard semen analysis with the most prevalent in recent literature evaluating the effects of sperm DNA fragmentation (SDF) on reproductive outcomes. A study of infertile males with normal semen parameters, yet failed IUI treatment despite an absence of a female factor, found that there was a significantly increased percentage of DNA damage present in the morphologically normal, motile spermatozoa of these men as compared to a fertile control [6]. These findings were corroborated in a later study reporting that 30% of infertile men with normal semen parameters were above the normal threshold for sperm DNA fragmentation [7]. Furthermore, several studies have supported that high DNA fragmentation levels hinder reproductive outcomes with IUI [8] and IVF [9] but with inconsistent effect on ICSI outcome [5]. Indeed, a recent guideline on the utility of DNA fragmentation testing in the clinical setting suggested that patients with poor IUI outcomes should be offered the option for SDF assessment as the results could expedite eventual treatment by IVF or ICSI [10]. More recent studies on the treatment of men with high SDF in their ejaculated spermatozoa experienced improved clinical outcome by using surgically retrieved spermatozoa, noted to have higher chromatin integrity [11, 12]. Thus, this simple test to screen the condition of the gamete genome is increasingly becoming recognized as a useful tool to assess the fertility of the male partner. However, it is still unclear to many investigators the best way to interpret this information and implement it into clinical practice [13].

The purpose of this study is to design a treatment algorithm based on sperm chromatin integrity to guide the management of couples with apparent unexplained infertility and that failed an IUI treatment. This approach may expedite treatment by steering a couple toward the most appropriate reproductive treatment option alleviating costs and minimizing patient distress.

## Material and methods

### Treatment algorithm

**Fig. 1:**
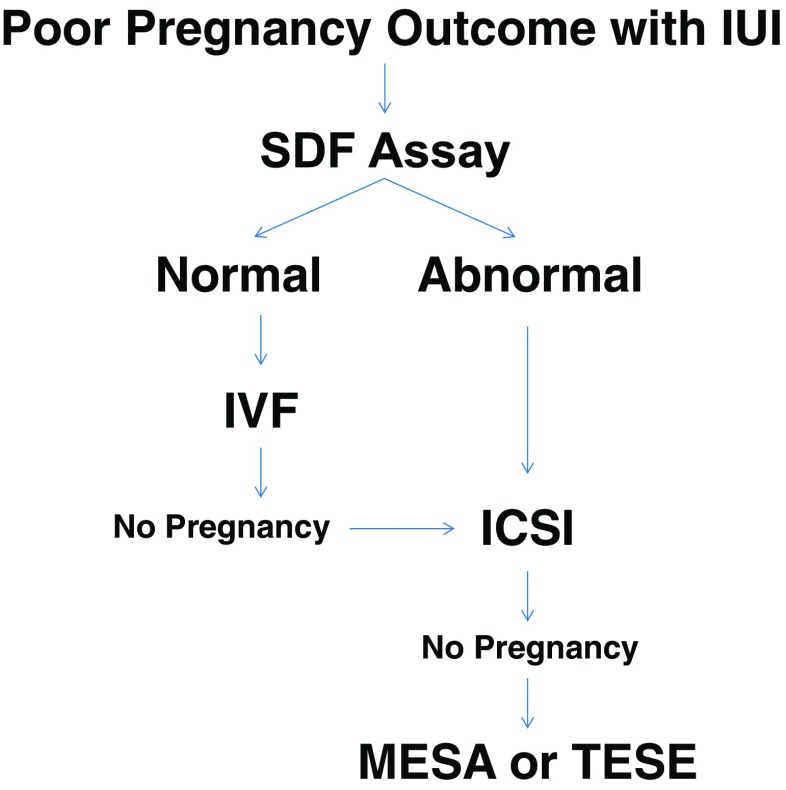
Flow chart depicting the treatment algorithm based on sperm chromatin integrity for couples with unexplained infertility. When couples with unexplained infertility failed to achieve pregnancy with IUI, the male partner is screened for chromatin integrity to assess SDF. Those with SDF below threshold are advised to undergo standard in vitro insemination. Couples with high DNA fragmentation in the ejaculate, instead were treated directly by ICSI. This option would also be available to couples that failed standard IVF. Couples with persistent SDF in their ejaculate and that failed ICSI were offered to undergo surgical sperm retrieval in their subsequent ICSI cycle

Couples with unexplained infertility that surprisingly resulted in a poor IUI outcome in repeated cycle were consented (Institutional Review Board of Weill Cornell Medicine IRB #1705018205 & IRB #1210013187) and included in a treatment algorithm (Fig. 1). Male partners were screened for genomic integrity using either the sperm chromatin structural assay (SCSA) or terminal deoxynucleotidyl transferase-mediated deoxyuridine triphosphate-fluorescein nick-end labeling (TUNEL) assay. If the SDF was within the normal limit, the couple was then treated by standard IVF. If the male partner presented with a compromised SDF, the preferential treatment was ICSI with ejaculated spermatozoa. This treatment was also offered to couples that had a normal SDF but failed to achieve pregnancy after standard in vitro insemination. If couples failed ICSI with ejaculated spermatozoa, they were then offered to undergo ICSI with surgically retrieved spermatozoa.

To further control for an eventual confounding female factor, a cohort of couples where the female was ≤ 35 years of age at the time of treatment was analyzed.

### Subjects

From January 2014 to May 2017, all couples that underwent sperm DNA fragmentation testing for unexplained infertility were included in the study. Couples were evaluated for unexplained infertility and diagnosed by a typical male and female workup resulting in a normal semen analysis for the male partner and a female with regular ovulation, tubal patency, and a normal uterine cavity yet still unable to conceive naturally after 1 year of attempts. Clinical outcome for intrauterine insemination, standard in vitro insemination, ICSI with ejaculated spermatozoa, and with surgically retrieved spermatozoa were recorded.

### Male gamete collection

Semen samples were collected by masturbation in sterile collection jars and were placed in an oven at 37 °C for 15 min to promote liquefaction. Upon initial analysis of the concentration and motility of the sample using 5 μL of raw semen in a Makler chamber, 20 μL of raw sample was set aside for smearing of slides for a TUNEL assessment or 200 μL was removed from the neat sample for patients scheduled for an SCSA. A standard semen analysis was performed on 1 mL of raw sample and results were reported comparing values to WHO 5th edition normal thresholds for volume (1.5–5.0 mL), concentration (≥ 15 million/mL), total motility (≥ 40%), progressive motility (≥ 32%), and normal morphology (≥ 4%) [4].

Surgical retrieval of spermatozoa was performed as per Schlegel [14]. Samples of the larger tubules were then dissected and dropped into a suspension, which passed through a 24-ga angiocatheter to be examined for the presence of spermatozoa on a glass slide under a phase-contrast microscope at × 200 magnification. If no spermatozoa were identified, more dissections were taken from the same testis and eventually the second. Surgical retrieval continued until spermatozoa were retrieved or there was concern that further dissection would be likely to compromise blood supply to the testicles. Samples of tissue without immediately apparent spermatozoa extraction were digested using collagenase.

### DNA fragmentation assays

Sperm Chromatin Structural Assay (SCSA) testing for chromatin integrity was performed by an outside laboratory (SCSA Diagnostics, Brookings, South Dakota, USA). Post-liquefaction, 200 μL of raw semen was removed from the neat samples and pipetted into a cryopreservation vial. The vial for an individual patient was flash frozen in liquid nitrogen and shipped to the outside facility for testing. A DNA fragmentation index value of < 25% was considered to be below threshold [15].

Terminal deoxynucleotidyl transferase-mediated deoxyuridine triphosphate-fluorescein nick-end labeling (TUNEL) assay protocol used for the TUNEL assay has been previously described by Palermo et al., 2014 [16]. Briefly, the assay was carried out using a commercially available kit (in situ cell death detection kit; Roche Diagnostics, Rotkreuz, Switzerland). Upon production, raw semen samples were placed in an oven at 37 °C for 15 min to encourage liquefaction. After which, glass slides were smeared with 5 μL of the semen sample and left to dry overnight. Fixation was carried out by placing slides in 4% paraformaldehyde for 1 h. Slides were then washed in PBS and left to dry overnight once more. Permeabilization was performed by exposing slides to 0.1% Triton X-100 and 0.1% sodium citrate in PBS for 2 min at 4 °C. Slides were washed thrice in PBS and air dried. The kit reagent was applied to the slides according to the specified dilution in the kit protocol and left to incubate with coverslips added in a humidified chamber at 37 °C for 1 h. Slides were subsequently washed thrice in PBS and DAPI Antifade was added in order to visualize nuclei of spermatozoa, which were then viewed under a fluorescent microscope for a fluorescent signal indicating DNA breakage. A minimum of 500 spermatozoa were assessed per patient with a sperm DNA fragmentation (SDF) of ≤ 15% considered to be normal.

### Reproductive treatments

Patients prepared for IUI treatment by completing ovarian superovulation by taking clomiphene citrate or gonadotropins daily for 5 days. Response and endometrial thickness were monitored by serial transvaginal ultrasounds. Estradiol and LH levels were also measured by serum hormone assays. Patients without an LH surge had their ovulation triggered by the use of 10,000 IU hCG when the dominant follicles reached 20 mm in diameter. Within 24 h of hCG injection, semen samples were collected by masturbation after 2–5 days of abstinence and allowed to stand at 35 °C to promote liquefaction. Concentration and motility were analyzed by viewing 5 μL of the semen sample in a Makler chamber. The sample was then combined with media comprised of HEPES-buffered human tubal fluid (H-HTF; Irvine Scientific, CA, USA) supplemented with human serum albumin (HSA solution G Series culture media; Vitrolife, Goteborg, Sweden) and centrifuged at 600*g* for 10 min. The pellet was then resuspended and evenly layered onto density gradient (Enhance-S Plus Cell Isolation Media, 90%; Vitrolife, Goteborg, Sweden) and centrifuged a second time at 300*g*. Afterwards, the bottom layer containing the motile spermatozoa was aspirated using a glass Pasteur pipette and resuspended in medium for a final centrifugation at 600*g* for 10 min in order to remove the silica gel particles from the sample. The sample was brought down to a volume of 0.5 mL for fresh and 0.4 mL for frozen samples and resuspended. The sample was finally assessed for concentration and motility and used for insemination.

### Stimulation protocols, oocyte retrieval, and embryo transfer

Stimulation protocol was determined by a careful consideration of multiple factors. Patient weight, age, serum anti-Mullerian hormone (AMH) level, antral follicular count, and patient response to prior stimulation protocols led the reproductive physician to create a plan that would best benefit the patient. Patients were treated with gonadotropins daily (Gonal F, EMD Serono, Geneva, Switzerland; Menopur, Ferring Pharmaceuticals Inc., Parsippany, NJ, USA; and/or Follistim, Merck, Kenilworth NJ, USA). A GnRH-antagonist (Ganirelix acetate, Merck, Kenilworth, NJ, USA; or Cetrotide, EMD Serono Inc., Rockland, MA, USA) or GnRH-agonist (leuprolide acetate, Abbott Laboratories, Chicago, IL, USA) was administered to suppress the function of their pituitary glands. When the diameters of the two leading follicles reached or surpassed 17 mm, the patient was triggered with human chorionic gonadotropin (hCG, Ovidrel, EMD Serono). Oocyte retrieval was performed under conscious sedation between 35 and 37 h after administering hCG. After either standard IVF or ICSI, embryos were cultured until the embryo transfer, which for the vast majority of patients (91%) occurred on day 3 post-insemination.

### Statistical analysis

Statistical analyses were performed on the study cohort characteristics as well as clinical outcomes. Continuous variables were described as a mean ± SD (standard deviation) and analyzed using Student’s *t* test. Categorical variables between groups, represented as a percentage, were assessed using the chi-square test. A *P* value was reported when considered significant at ≤ 0.05.

## Results

Couples that failed repeated IUI attempts (*n* = 354) with normal semen parameters (concentration = 49.0 ± 28 × 10^6^/mL, motility = 48.6 ± 9%, morphology = 4.3 ± 1%) underwent chromatin fragmentation testing for a perceived, unspecified male factor infertility. The algorithm treatment route for these couples can be referenced in Fig. 1. The average pregnancy rate within these couples was 1.8%, significantly lower than the 23.9% observed in our average IUI population with normal semen parameters. Of these couples, 31 couples with normal SDF (9.8 ± 4.6% as assessed by SCSA, 11.8 ± 6.2% as assessed by TUNEL) underwent standard in vitro insemination (Fig. 1), reporting a clinical pregnancy rate of 12.7%. Of the remaining couples (*n* = 343), 90% had abnormal SDF and underwent ICSI with ejaculated spermatozoa (Fig. 1). This cohort also included couples that failed standard IVF and reported a cumulative pregnancy rate of 18.7%.

In a previous analysis, we assessed the chromatin fragmentation at different levels of the male genital tract in men that had an abnormally high DNA fragmentation in the ejaculate. The epididymal and testicular populations observed SDF rates that fell within the threshold considered to be normal by our standards. Therefore, in this study, couples that repeatedly failed ICSI with ejaculated spermatozoa, approximately 10%, underwent additional cycles of ICSI with surgically retrieved spermatozoa, yielding an overall clinical pregnancy rate of 31.0% (Fig. 1; Table 1).

**Table 1:** Characteristics and clinical outcome of couples with unexplained infertility allocated to different reproductive treatments

	IUI	IVF	ICSI	
			Ejaculated	Surgically retrieved
No. of patients	354	31	343	34
No. of cycles	1133	63	796	58
Male age (mean ± SD)	40.7 ± 6	39.4 ± 5	39.8 ± 6	45.6 ± 11
Female age (mean ± SD)	37.5 ± 5	36.3 ± 4	37.6 ± 4	37.4 ± 4
Fertilization (%)	–	425/696 (61.1)^a^	5210/7139 (73.0)^b^	354/533 (66.4)^c^
Clinical pregnancy (%)	20/1133 (1.8)^d^	8/63 (12.7)^e^	149/796 (18.7)^f^	20/58 (31.0)^g^
Implantation (%)	–	11/151 (7.3)^h^	178/1650 (10.8)^i^	25/95 (26.3)^j^
Delivery and ongoing (%)	14/1133 (1.2)^k^	6/63 (9.5)^l^	105/796 (13.2)^m^	18/58 (31.0)^n^

a vs b, c: *χ*^2^, 2 × 3, 2 *df*, effect of insemination method on fertilization rates, *P* < 0.00001; d vs e, f, g: *χ*^2^, 2 × 4, 3 *df*, effect of insemination method on clinical pregnancy rates, *P* < 0.00001; h vs i, j: *χ*^2^, 2 × 3, 2 *df*, effect of insemination method on implantation rates, *P* < 0.00001; k vs l, m, n: *χ*^2^, 2 × 4, 3 *df*, effect of insemination method on delivery and ongoing pregnancy rates, *P* < 0.00001

**Fig. 2:**
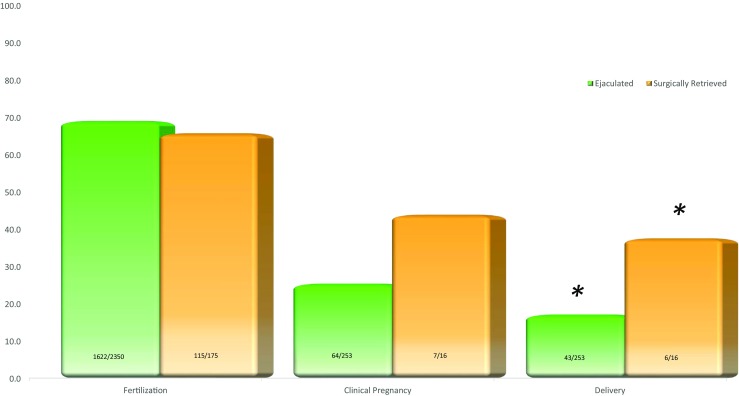
Clinical outcome of couples with high SDF undergoing ICSI with different sperm sources. Clinical outcome according to sperm source of couples where the female partner was ≤ 35 at the time of insemination and the men presented with persistently high SDF in the ejaculate. While fertilization and clinical pregnancy rate did not appear to differ in relation to sperm source, surgically retrieved spermatozoa yielded higher delivery and ongoing rates (*P < 0.05)

In order to exclude a contribution of a potential confounding female factor, we reassessed our data following the same treatment algorithm by considering female partners ≤ 35 years of age at the time of the latest treatment cycle. Indeed, the inclusion criteria were IUI couples with normal semen parameters and a female partner ≤ 35 years of age. The combined IUI cycles of these patients resulted in a clinical pregnancy rate of 2.9% (Fig. 1; Table 2). Those with normal SDF (TUNEL 9.1 ± 1% and SCSA 10.6 ± 6%) underwent standard in vitro insemination, and these cases achieved a clinical pregnancy rate (CPR) of 18.4% (Fig. 1). Couples that failed to achieve pregnancy through standard in vitro insemination together with those that had abnormal chromatin fragmentation were treated with ICSI using ejaculated spermatozoa resulting in a CPR of 25.3% (Fig. 1). Finally, 7.9% (10/127) of couples with repeated failure to obtain a successful pregnancy, characterized by a high SDF in the ejaculate as assessed by TUNEL (21.4 ± 6%; 16–31.8) and SCSA (24.8 ± 6%; 18–34), agreed to undergo surgical retrieval of spermatozoa. After successful surgical sperm retrieval, these couples underwent additional cycles of ICSI using epididymal or testicular spermatozoa with normal SDF and resulted in a CPR of 43.8% (Figs. 1 and 2). A detailed performance of the clinical outcomes of surgically retrieved specimen over the ejaculate is shown in Fig. 2.

**Table 2:** Characteristics and clinical outcome of couples with unexplained infertility whose female partner is ≤ 35 years old allocated to different reproductive treatments

	IUI	IVF	ICSI	
			Ejaculated	Surgically retrieved
No. of patients	133	16	127	10
No. of cycles	342	38	253	16
Male age (mean ± SD)	36.3 ± 4.3	37.2 ± 4	35.9 ± 4	34.2 ± 5
Female age (mean ± SD)	32.8 ± 2	33.9 ± 2	32.9 ± 2	32.0 ± 3
Fertilization (%)	–	289/420 (68.8)	1622/2350 (69.0)	115/175 (65.7)
Clinical pregnancy (%)	10/342 (2.9)^a^	7/38 (18.4)^b^	64/253 (25.3)^c^	7/16 (43.8)^d^
Implantation (%)	–	10/87 (11.5)^e^	81/427 (19.0)^f^	9/21 (42.9)^g^
Delivery and ongoing (%)	7/342 (2.0)^h^	5/38 (13.2)^i^	43/253 (17.0)^j^	6/16 (37.5)^k^

a vs b, c, d: *χ*^2^, 2 × 4, 3 *df*, effect of insemination procedure on clinical pregnancy rates, *P* < 0.00001; e vs f, g: *χ*^2^, 2 × 3, 2 *df*, effect of insemination procedure on implantation rates, *P* < 0.005; h vs i, j, k: *χ*^2^, 2 × 4, 3 *df*, effect of insemination method on delivery and ongoing pregnancy rates, *P* < 0.0001

## Discussion

We attempted to integrate an additional screening tool into the male fertility work-up in order to complement the semen analysis, which provides no effective information on sperm competence. Although it is apparent that there are many potential underlying causes of the failure for these couples to conceive, testing the male partner for SDF can reveal an affliction of the male gamete’s genomic integrity that hinders chances of conception. Thus, we propose to measure the integrity of the male genome in couples where semen analysis appears uninformative, and that have failed to achieve pregnancy with IUI even with a young female partner as other authors have suggested [7].

DNA fragmentation assays are becoming widely popular among reproductive centers and a large amount of data is now available. These various assays have untested thresholds and often lack concordance among each other [17]. Among these tests, SCSA is considered the gold standard [18] and the COMET assay is reputed to be the most sensitive and particularly useful for identifying double-stranded breaks [19] although recent meta-analysis studies have reported the TUNEL assay as having high sensitivity and specificity compared to other DNA fragmentation assays [17, 20]. A simple and more consistent approach is to utilize in-house testing that would allow consistent, expedited, and cost-effective methods to screen couples.

The presence of a normal SDF would suggest the use of standard in vitro insemination to enhance chances of pregnancy (*P* = 0.01) while couples that present with abnormal SDF should benefit directly from ICSI to alleviate the effect of a compromised male genome, as ICSI would select the most motile spermatozoon [21]. Indeed, the correlation of SDF with motility has been known for some time [16] and therefore, would be advantageous to use ICSI insemination [22]. It is, indeed, the proportion of highly progressive motile spermatozoa in a specimen that indicates low incidence of SDF and therefore, techniques that select for the most motile spermatozoa for injection, such as ICSI, are the preferred method [16, 23]. Moreover, couples with normal SDF that had failed IVF may have better chances of pregnancy with ICSI using ejaculated spermatozoa [24, 25]. It is becoming more clinically relevant to propose to couples with unexplained infertility or with recurrent pregnancy loss the utilization of surgically retrieved spermatozoa. It has been observed in several studies, including our own, that the collection of spermatozoa from the epididymis or more preferably the testicle, displays SDF below threshold even in men with high DNA fragmentation in their ejaculate [10, 11, 26].

The situation becomes more complex when the couple fails ICSI even with ejaculated spermatozoa. Our intimate collaboration with the reproductive urologists has allowed us to perform a study on the usefulness of utilizing surgically retrieved spermatozoa in men with higher incidence of chromatin fragmentation in their ejaculate. Our experience has evidenced that even in the most severe cases of DNA fragmentation, the incidence of SDF progressively decreases when samples are retrieved from the proximal sites of the male genital tract, resulting in almost consistently normal SDF in the testicle. When comparing semen source, there is a clear improvement in clinical outcome utilizing testicular and epididymal spermatozoa in comparison to the ejaculated counterpart, supporting the concept that sperm chromatin integrity degrades post-testis in transit through the male genital tract [12]. While the option of repeating an ICSI attempt with ejaculated spermatozoa is still valid, the decision to use surgically retrieved spermatozoa is becoming more accepted. Indeed, recent studies have shown higher clinical pregnancy and delivery rates with reduced miscarriages [11, 12] supported by the reassuring preliminary data on the perinatal outcomes of newborns resulting from testicular spermatozoa [27, 28].

This study delineates how testing for sperm DNA fragmentation can potentially assist the treating physician in determining the best reproductive treatment method for an individual couple based on their infertility profile. The use of a supplemental assay to assess the male gamete beyond a standard semen analysis is an affordable and reasonable step to take prior to choosing the next treatment approach, potentially decreasing the number of unnecessary treatment cycles. This may alleviate some financial and emotional distress and ultimately shorten the time to conceive.
